# Effect of nonsurgical periodontal therapy on the eradication of gastric *Helicobacter pylori* infection: A randomized clinical trial

**DOI:** 10.34172/japid.025.3895

**Published:** 2025-08-06

**Authors:** Parichehr Behfarnia, Reza Birang, Shirin Rostami, Vahid Sebghatollahi

**Affiliations:** ^1^Department of Periodontics, Dental Implant Research Center, Dental Research Institute, School of Dentistry, Isfahan University of Medical Sciences, Isfahan, Iran; ^2^Department of Periodontics, School of Dentistry, Isfahan University of Medical Sciences, Isfahan, Iran; ^3^Department of Internal Medicine, School of Medicine, AL-Zahra Hospital, Isfahan University of Medical Sciences, Isfahan, Iran

**Keywords:** Helicobacter pylori, Nonsurgical periodontal debridement, Peptic ulcer, Scaling and root planing, Therapy

## Abstract

**Background.:**

This study aimed to assess the effectiveness of "a combined systemic and nonsurgical periodontal treatment" in eradicating gastric *Helicobacter pylori* in patients having the bacterium within their subgingival biofilm.

**Methods.:**

This randomized clinical trial (RCT) investigated 102 patients diagnosed with peptic ulcer or dyspepsia and a positive stomach test for *H. pylori* infection (G+). Participants with a negative test for oral infection received only triple therapy (G3, n=38), and those positive for oral infection were randomly allocated to one of the two treatment regimens: a 14-day course of triple therapy (comprising antibiotics, antimicrobials, and proton pump inhibitors) alongside periodontal therapy (G1, n=32) or triple therapy alone (G2, n=32). The effectiveness of *H. pylori* eradication was assessed four weeks after treatment using the *H. pylori* stool antigen (stool Ag) test. Data analysis was performed using SPSS 22.

**Results.:**

In the G2 and G3 groups, triple therapy achieved success rates of 52% and 84%, respectively. When periodontal therapy was integrated with triple therapy in the G1 group, the success rate was 80%. Significant differences were observed between the G1 and G2 groups (*P*=0.037) and also between the G3 and G2 groups (*P*=0.015). Conversely, no significant difference was found between the G1 and G3 groups (*P*>0.05).

**Conclusion.:**

Periodontal therapy has the potential to substantially increase the efficacy of *H. pylori* eradication regimens for gastric infections.

## Introduction

*Helicobacter pylori*, a bacterium that inhabits the gastric mucosa, is a significant human pathogen. Its presence is strongly linked to the development of chronic gastritis, peptic ulcer, gastric adenocarcinoma, and mucosa-associated lymphoid tissue (MALT) lymphoma.^1^ Eradicating *H. pylori* promotes gastric ulcer healing and diminishes both the recurrence of ulcers and the incidence of associated gastrointestinal diseases.^2^ H. *pylori* is present in the stomachs of roughly half the global population.^3^ Nevertheless, the precise mechanisms by which this microorganism is transmitted between individuals remain undefined.

*H. pylori* can be transmitted through various pathways, including iatrogenic means, fecal-oral contact, oral-oral contact, and via contaminated food and water.^4^ In recent decades, the rate at which *H. pylori* infection is eradicated has significantly declined. There is also a high likelihood of this rate dropping even further due to the increasing issue of drug resistance.^5^ The recurrence rate of *H. pylori* infection varies considerably, ranging from 0% to 41.5%.^6^ Evidence suggests that the highest incidence of infection recurrence occurs within the first year following treatment, strongly indicating that these cases are more likely attributable to re-infection rather than recrudescence.^7^*H. pylori* has been detected in various oral niches, including saliva, dental plaque, and gingival pockets. Research indicates a wide range in the reported prevalence of *H. pylori* in dental plaque, varying from 0% to 100% across different studies.^8^ Furthermore, genotypic analysis of *H. pylori* isolated from disparate sites reveals identical or highly similar strains, suggesting either the presence of a single species or genetic mutations within the same species.^9,10^

 In research conducted by Hu et al^11^ and Gao et al,^12^ polymerase chain reaction (PCR) analysis consistently demonstrated that dental plaque served as the primary reservoir for this bacterium, with higher concentrations observed in subgingival plaque compared to supragingival plaque. Furthermore, the presence of urea within biofilms enhances the viability of urease-producing bacteria like *H. pylori*, with a synergistic relationship possibly accounting for the elevated prevalence of this bacterium in dental plaque relative to other oral sites.^13^

 Multiple studies have indicated a positive correlation between the levels of dental plaque and oral hygiene, and the presence of *H. pylori* infection in both the oral cavity and the gastric system.^14^ The predilection of *H. pylori* for supragingival plaque can be attributed to its microaerophilic nature. *H. pylori* present in supragingival plaque can migrate to the subgingival plaque, with elevated levels of the bacterium observed in both locations in individuals with periodontal diseases.^15^ The persistence of *H. pylori* within the oral cavity is contingent upon its interactions with other microorganisms in the plaque biofilm. *H. pylori* demonstrates selective adhesion to certain bacterial species, including *Fusobacterium nucleatum* (*F. nucleatum*),^16,17^ *Tannerella forsythia*,^18^ and *Porphyromonas gingivalis*.^19^ Given the proliferation of these bacteria in individuals with periodontal disease, it is plausible that dental plaque in these patients serves as a reservoir for *H. pylori*, likely due to its interactions with these cohabiting bacterial species.^8^ Periodontal diseases may be linked to gastrointestinal infections and their recurrence; however, the precise involvement of the oral cavity in the transmission and relapse of these infections remains a subject of ongoing debate.

 Triple therapy, the most common treatment for *H. pylori* infection,^20-23^ demonstrates limited efficacy in eradicating oral *H. pylori*.^24-26^ Since dental plaque is a bacterial biofilm, it effectively shields the resident microorganisms from systemic antimicrobial agents,^8^ thereby impeding antibiotic penetration into the biofilm structure.^27^ The diminished concentration of antibiotics in saliva, compared to gastric levels, consequently attenuates their efficacy even against microorganisms present in non-plaque environments, such as saliva itself.^28^

 The presence of *H. pylori* in the oral cavity has been linked to a diminished efficacy of eradication therapy, potentially leading to treatment failure and recurrent gastrointestinal infections.^8^ Research has explored the impact of periodontal therapy on gastric *H. pylori* infections. For instance, Sheu et al^29^ proposed that individuals with periodontal disease may be more susceptible to *H. pylori* recurrence, even following successful eradication of the bacterium. Some researchers have suggested that integrating periodontal therapy with standard triple therapy significantly improves the eradication of gastrointestinal *H. pylori* infection. This combined approach is more effective than medication alone, culminating in a notably lower recurrence rate of the infection among patients who undergo periodontal therapy.^30-32^ The oral cavity’s potential to harbor and transmit gastrointestinal infections suggests that periodontal therapy could be a valuable intervention in treating gastric *H. pylori *infections.^33-35^

 Given the ongoing debate surrounding the involvement of oral *H. pylori* in gastrointestinal infections and the effectiveness of periodontal therapy in eradicating gastric *H. pylori*, this study aimed to assess the efficacy of nonsurgical periodontal therapy in eradicating *H. pylori* infection.

## Methods

 This study, performed at the Department of Periodontics (Isfahan, Iran), received ethical approval from Isfahan University of Medical Sciences (Approval ID: IR.MUI.REC.1396.3.174) and was registered with the Iranian Registry of Clinical Trials (IRCT) (Approval ID: IRCT20150210021029N3). All participants provided written informed consent, and their involvement was voluntary.

###  Study design

**Figure 1:**
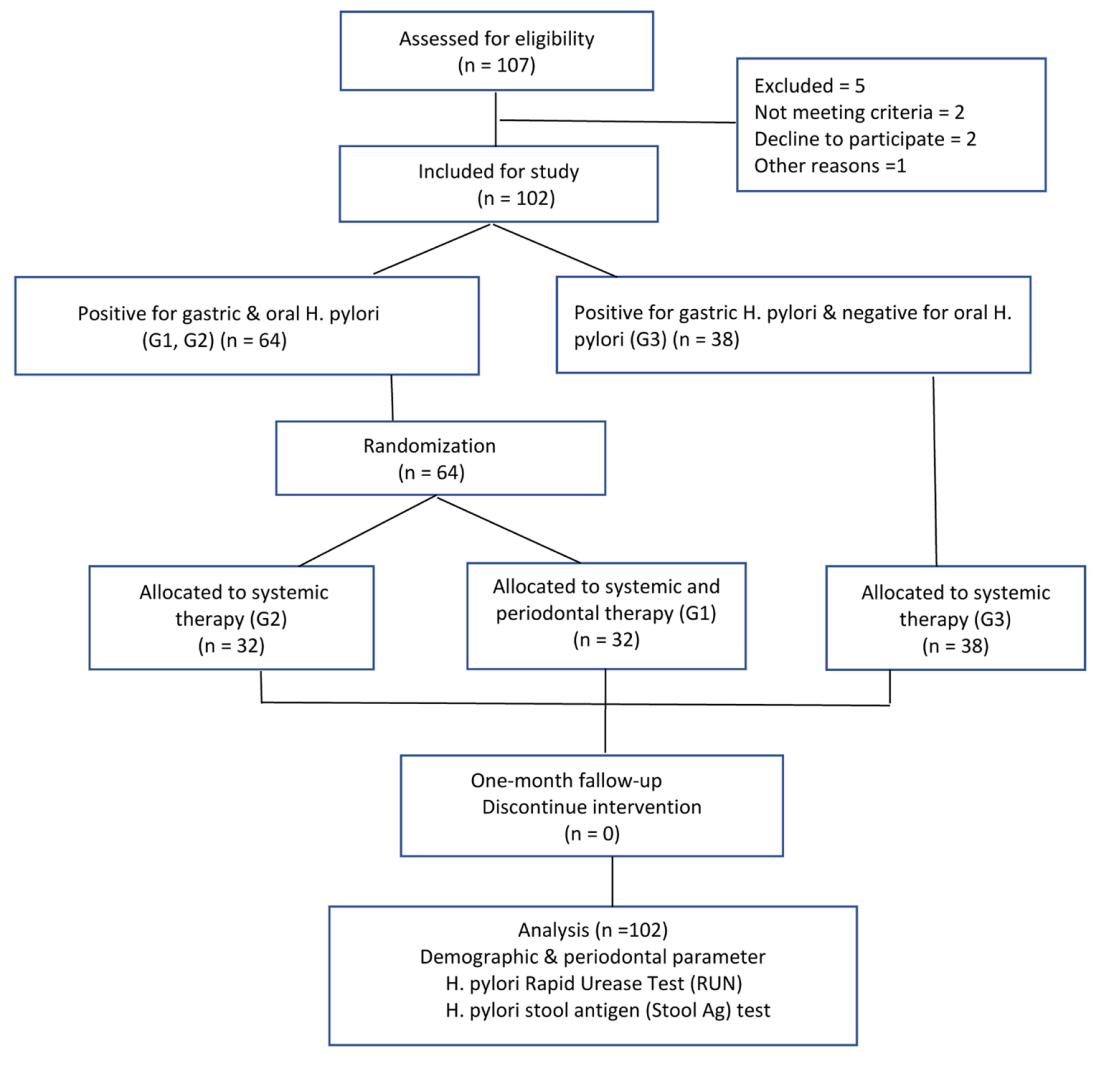
Flow diagram of the study

 The current single-center, double-masked, prospective, randomized clinical trial (RCT) was conducted with a parallel-group design. Figure 1 illustrates the methodological workflow. This study was a double-blind RCT, with a minimum of 25 patients in each of the study groups, with an 80% probability that a difference of 0.37 would be significant at the α = 0.05 level. Initially, 107 patients were recruited. Five participants subsequently dropped out, resulting in a final 102 patients. These patients, diagnosed with peptic ulcer or dyspepsia, tested positive for gastric infection via serology or biopsy. They were referred from the gastrointestinal clinic of Al-Zahra and provided informed consent before receiving treatment. The reporting of results conformed to the guidelines outlined in the 2010 Consolidated Standards of Reporting Trials (CONSORT) statement.

###  Selection criteria

 The inclusion criteria encompassed patients diagnosed with peptic ulcer or dyspepsia, who exhibited positive serological or biopsy results for gastric infection.

 The exclusion criteria for this study included recent treatment for *H. pylori* within the preceding 2 months, the use of antibiotics or gastrointestinal medications (e.g., proton pump inhibitors) in the preceding 2 months, prior gastric surgery, and a history of periodontal therapy within the preceding 6 months. Upon commencing the study, each patient’s questionnaire was used to document demographic data, including age, gender, smoking status, and alcohol use. Subsequently, a comprehensive full-mouth periodontal examination was conducted for all participants. An investigator (Sh. R.) meticulously evaluated several periodontal indicators, including the plaque index, bleeding on probing, presence of calculus, coated tongue, and clinical probing depth.^36^ Subsequently, the subgingival plaque was analyzed for the presence of *H. pylori* using the rapid urease test (RUT).^35^ This diagnostic procedure, detailed elsewhere, was conducted using the RUT kit (Bahar Medical Laboratory, Tehran, Iran). Subgingival plaque was meticulously collected from 3‒4-mm periodontal pockets of the posterior teeth using a Williams periodontal probe. The collected plaque was then transferred into a tube containing 0.5 mL of solution. A positive test result was indicated by a color transformation of the solution from yellow to pink within 20 minutes.

 While various oral microorganisms, including *Streptococcus species*, *Haemophilus species*, and *Actinomyces species*, possess the capacity for urea production, only *H. pylori* synthesizes urea in quantities substantial enough to induce a color change in solution within 20 minutes.^20^

###  Interventions

 In a study involving 64 participants who tested positive for an RUT, individuals were randomly divided into two distinct groups using random allocation software. Group 1, designated as G1 (G ^+^ O ^+^ tP), comprised participants found to be positive for *H. pylori* in both gastric and oral samples. This group received a combination of triple eradication therapy and nonsurgical periodontal therapy. Group 2, designated as G2 (G ^+^ O ^+^ t), comprised participants who tested positive for *H. pylori* in both gastric and oral specimens and received triple eradication therapy exclusively. Additionally, 38 participants who yielded negative RUT results were allocated to group 3 [G3 (G ^+^ O - t)]; these individuals tested positive for *H. pylori* in gastric samples but were negative in oral samples and also underwent triple eradication therapy.

###  Treatment regimens

 In all the three groups, the triple therapy regimen comprised 40 mg of pantoprazole administered twice daily, 240 mg of bismuth administered twice daily, 1 g of amoxicillin twice daily, and 500 mg of clarithromycin administered twice daily, sustained over 14 days. For G1, periodontal therapy involved comprehensive oral hygiene instruction and full-mouth disinfection (FMD). This FMD procedure encompassed a single-stage, complete scaling and root planing executed with an ultrasonic device, alongside the irrigation of periodontal pockets, the tongue, and the tonsils using a 0.2% chlorhexidine-gluconate solution (chlorhexidine-Najo, Tehran, Iran) concurrent with the triple therapy.

 Following this, an 0.2% chlorhexidine gluconate mouthwash was prescribed twice daily for two weeks. Concurrently, periodontal therapy commenced alongside triple therapy. To assess the impact of these treatments on *H. pylori* eradication rates, a stool antigen (stool Ag) test was conducted at the Al-Zahra Hospital laboratory at least one month after treatment.

###  Outcomes

 This study primarily aimed to evaluate the efficacy of a combined nonsurgical periodontal and systemic therapeutic approach compared to systemic therapy alone for eradicating *H. pylori* from the subgingival biofilm in patients harboring the bacterium.

###  Randomization and concealment

 Sixty-four participants, all testing positive for RUT, were randomly allocated to one of two groups (G1 and G2) using random allocation software. G1 underwent systemic and nonsurgical periodontal therapy (G ^+^ O ^+^ tp), while G2 received systemic therapy only (G ^+^ O ^+^ t).

###  Blinding 

 The researcher responsible for outcome evaluation and statistical analysis was blinded.

###  Statistical analysis

 Data analysis was conducted using SPSS 22. Statistical significance was defined at *P* < 0.05.

## Results

 In this study, 102 participants were enrolled, comprising 49 males (48%) and 53 females (52%). A chi-squared test revealed no significant difference in gender distribution across the groups (*P* = 0.236). Among the participants, 16.2% were identified as smokers. Fisher’s exact test revealed no significant difference in smoking prevalence across the three groups (*P* = 0.799). Additionally, 9.3% of participants reported alcohol use, with Fisher’s exact test indicating no significant differences across the three groups (*P* = 0.99). Dental calculus was observed in 44 patients (58.7%). A chi-squared test revealed no significant difference in the prevalence of dental calculus across the three study groups (*P* = 0.113). A coated tongue was present in 14 patients (18.7%). Fisher’s exact test indicated no significant difference in the occurrence of coated tongue across the three groups (*P* = 0.99). Furthermore, a Kruskal-Wallis test demonstrated no significant difference in the mean number of amalgam restorations across the three study groups (*P* = 0.839) (Table 1).

 The mean plaque index measurements for the three groups were as follows: G1: 66.28 ± 15.83, G2: 63.61 ± 19.49, G3: 63.6 ± 18.6. The highest mean plaque index was observed in G1, while G2 exhibited the lowest. However, a one-way analysis of variance (ANOVA) revealed no significant difference in the mean plaque index across the three groups (P = 0.8).

 The mean plaque index was similar across the groups with (64.72 ± 17.67) and without (63.6 ± 18.16) oral *H. pylori*. An independent samples t-test was employed to analyze these plaque index data between the two groups. The findings revealed no significant difference between the two groups, those with and those without oral *H. pylori* (*P* = 0.798). The mean probing depth measurements were 2.51 ± 0.8 in G1, 2.79 ± 1.13 in G2, and 2.62 ± 0.42 in G3.

 The Kruskal-Wallis test results indicated no significant difference in the mean scores of the three groups (*P* = 0.669). Additionally, the mean probing depth was recorded as 2.654 mm in the group with oral *H. pylori* and 2.625 mm in the group without oral *H. pylori*. Consistent with these findings, the Mann-Whitney test revealed no significant difference in mean probing depth between individuals with and without oral *H. pylori* (*P* = 0.496).

 In G1, one month after treatment, the RUT yielded negative results in 14 patients (56%), while the remaining 11 patients (44%) continued to test positive. A McNemar test was conducted to compare the primary and secondary RUT results, revealing a significant difference (*P* < 0.001).

**Figure 2:**
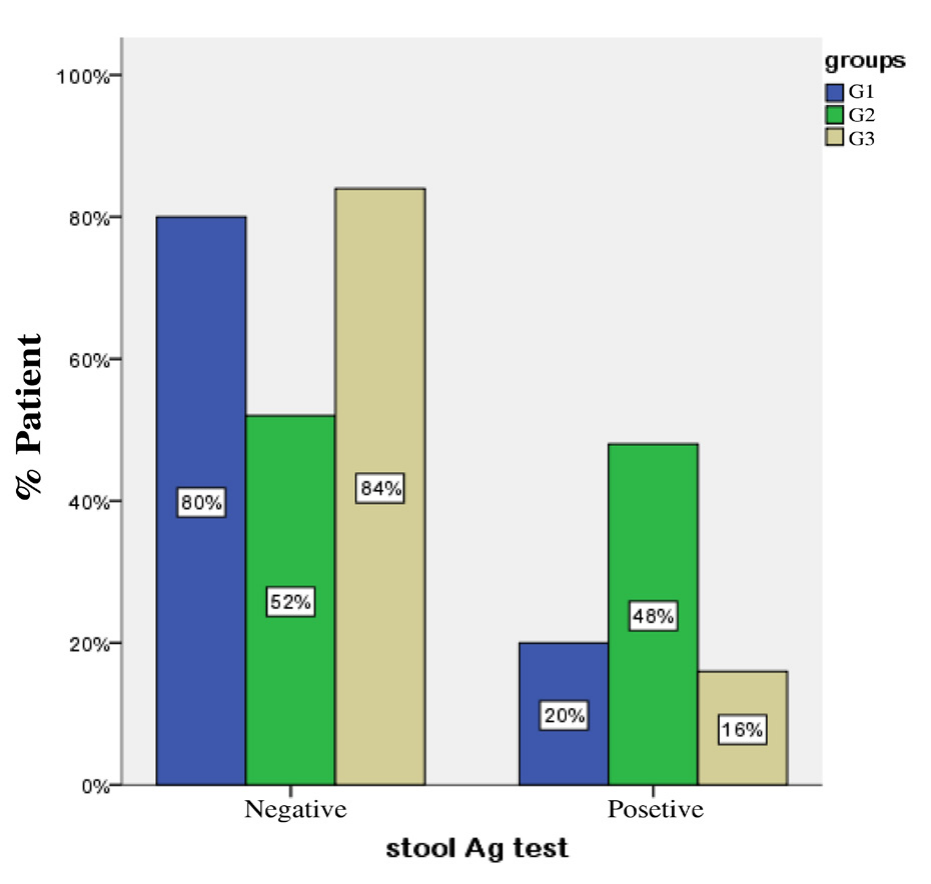
Percentage of patients with negative or positive stool Ag test one month after treatment. G1: positive for *H. pylori* in both gastric and oral samples and received combined treatment, G2: positive for *H. pylori* in both gastric and oral samples and received triple therapy alone, G3: positive for *H. pylori* in gastric but negative in oral samples and received triple therapy. Stool Ag test: EIA based on enzyme immune assay

 One month following drug administration, positive stool Ag results were observed in 6 subjects from G1, 15 subjects from G2, and 6 subjects from G3. The corresponding eradication rates for these groups were 80%, 52%, and 84%, respectively (Figure 2).

 A chi-squared test revealed significant differences between G1 and G2 (*P =*0.037) and between G2 and G3 (*P =*0.015). However, Fisher’s exact test results revealed no significant difference (*P =*1.000) between G1 and G3.

**Table 1:** Demographic and clinical parameters of all study subjects

**Demographic and clinical parameters**	** G1** ^*^	** G2**	** G3**	***P*** ** value**
Male	17	14	18	0.236
Female	18	19	16	
Smoker^**^	8	6	7	0.799
Alcohol user^**^	2	2	3	0.99
Calculus^#^	18	15	11	0.113
Coated tongue^#^	4	5	5	0.99
Amalgam^##^	5.6	5.76	6.48	0.839

*Groups: G1 positive for *H. pylori* in both gastric and oral samples and received combined treatment; G2 positive for *H. pylori* in both gastric and oral specimens and received triple therapy; G3 positive for *H. pylori* in gastric but negative in oral samples and received triple therapy. **Number of smokers and alcoholic patients in each group. #Number of patients who have calculus or a coated tongue. ##Mean number of amalgam restorations in each group.

## Discussion

 This research aimed to evaluate the impact of nonsurgical periodontal therapy on the eradication of gastric *H. pylori*. Our findings indicated that 80% of patients receiving both systemic triple therapy and nonsurgical periodontal therapy achieved gastric eradication, whereas only 52% of patients undergoing systemic triple therapy alone demonstrated comparable results. The combined therapy group showed an enhanced clinical outcome, achieving statistical significance. This finding aligns with prior research,^30,31,37,38^indicating that periodontal therapy positively impacts the healing of gastrointestinal infections. However, this observation diverges from the conclusions drawn by Zaric et al^34^ and Song and Li.^39^ In Zaric et al’s study, the G ^+^ O ^+^ group exhibited elevated mean plaque index and probing depth compared to the G ^+^ O - group. However, this observed difference was not statistically significant (*P* = 0.07). These results imply that *H. pylori* may preferentially colonize newly formed plaque, while established or older plaque demonstrates less susceptibility to colonization.^34^ The current understanding suggests that the specific composition of dental plaque containing *H. pylori* holds greater significance than the sheer volume of plaque accumulation. Probing depth appears to be a potential risk factor for the presence of oral *H. pylori*, which is likely due to the interaction of *H. pylori* with enhanced periopathogens, such as *F. nucleatum*, in deeper periodontal pockets.^16,17^ The discrepancies observed between studies, despite similar sample sizes, may stem from variations in the methodologies employed for *H. pylori* detection. For instance, Zaricet al’s study used PCR and the urea breath test (UBT) to identify both oral and digestive *H. pylori* bacteria.^34^ In contrast, the current study relied on the RUT and stool Ag detection methods. *H. pylori* is frequently detected in dental plaque. Therefore, nonsurgical periodontal therapy, which involves the removal of plaque and calculus from tooth surfaces, may be a significant factor in both the eradication and prevention of gastrointestinal *H. pylori* re-infection.

 As suggested by Sheu et al,^29^ periodontal disease may increase an individual’s susceptibility to recurrent *H. pylori* infection, even following successful eradication of the bacterium. Furthermore, studies by Gao et al^12^ and Butt et al^31^ have demonstrated that a combined approach of drug therapy and periodontal therapy is more effective in eradicating gastrointestinal *H. pylori* infections compared to drug therapy alone. Their research also suggests that individuals with oral microorganisms exhibit a higher rate of infection recurrence. Namiot et al^33^ presented findings that diverge from other research, indicating no improvement in the effectiveness of *H. pylori* eradication in the gastrointestinal tract through enhanced oral health and hygiene.This discrepancy may stem from two key factors: First, their intervention was limited to mechanical oral hygiene alongside drug therapy, and second, the study did not assess the presence of oral *H. pylori* in the participant groups.^33^

 The differential success rates observed in *H. pylori* eradication—specifically, the efficacy of combined medication and periodontal therapy in patients with oral *H. pylori*, versus the success of drug therapy alone in those without oral *H. pylori* suggest a critical insight. These findings collectively imply that the presence of oral *H. pylori* may serve as a potential risk factor for the failure of conventional drug therapy aimed at eradicating *H. pylori*. Dental plaque serves as a natural host for *H. pylori*, potentially contributing to the recurrence of infections following gastric eradication therapy. Therefore, screening patients for *H. pylori* within dental plaque could aid in its elimination and reduce the development of antibiotic resistance. This finding aligns with studies indicating a concurrent eradication of *H. pylori* in both oral and gastric sites among patients.^39,40^

 While systemic therapy effectively eradicates *H. pylori* from the stomach, the bacterium residing in dental plaque is shielded from systemic antibiotics by its protective biofilm. Consequently, if not mechanically removed, this dental plaque acts as a potential reservoir for gastric re-infection.

 Certain research suggests a correlation between the existence and intensity of periodontal disease and the concentration of *H. pylori* in the oral cavity. Given the challenges in eradicating oral *H. pylori* through drug therapy and the potential for dental plaque to serve as a reservoir for infection recurrence, periodontal treatment may effectively diminish oral *H. pylori* levels. In contrast, drug therapy alone has not demonstrated a significant reduction. The presence of *H. pylori* within dental plaque suggests that disrupting this plaque through mechanical and chemical interventions is crucial for its eradication. Current research indicates that antimicrobial agents are unlikely to be effective against *H. pylori* in this context. The plaque’s structural integrity must first be disrupted for these agents to be effective.^40^ A limitation of this study is its limited sample size and brief follow-up period; thus, further research, ideally in the form of large-scale RCTs, is required to corroborate the efficacy of nonsurgical periodontal therapy in *H. pylori* management.

## Conclusion

 The current research demonstrated that while antibiotic therapy alone effectively eliminated gastrointestinal infections, incorporating periodontal therapy into standard therapies for oral *H. pylori* patients more significantly reduced both oral and gastrointestinal *H. pylori* load. Given that the periodontal pocket serves as a primary reservoir for *H. pylori*, the observed effect likely stems from a reduction in both plaque accumulation and probing pocket depth.

## Competing Interests

 The authors declare that they have no competing interests.

## Consent for Publication

 Not applicable.

## Data Availability Statement

 All data regarding the methodology of the manuscript have been shared.

## Ethical Approval

 The present study was approved by the Ethics Committee of Isfahan University of Medical Sciences (Code: ID: IR.MUI.REC.1396.3.174).
